# The impact of JAK2V617F mutation on Philadelphia-negative myeloproliferative neoplasms

**DOI:** 10.3906/sag-2103-247

**Published:** 2021-09-04

**Authors:** Ezgi ŞAHİN, İpek YÖNAL-HİNDİLERDEN, Fehmi HİNDİLERDEN, Aynur ADAY, Meliha NALÇACI

**Affiliations:** 1 Division of Hematology, Department of Internal Medicine, Faculty of Medicine, İstanbul University, İstanbul, Turkey; 2 Division of Hematology, Department of Internal Medicine, Hamidiye Faculty of Medicine, University of Health Sciences, İstanbul, Turkey; 3 Division of Medical Genetics, Department of Internal Medicine, Faculty of Medicine, İstanbul University, İstanbul, Turkey

**Keywords:** JAK2V617F mutation, polycthemia vera, essential thrombocythemia, primary myelofibrosis, Philadelphia-negative myeloproliferative neoplasms

## Abstract

**Background/aim:**

JAK2V617F mutation is expressed in almost all polycthemia vera (PV), 55% of essential thrombocythemia (ET), and 65% of primary myelofibrosis (PMF) patients. Studies investigating phenotypic effects of JAK2V617F mutation on Philadelphia-negative myeloproliferative neoplasms (Ph-negative MPNs) have reported controversial results. This study aims to determine the impact of JAK2V617F mutation on clinical phenotype and outcome in Ph-negative MPNs.

**Materials and methods:**

Clinical correlates and long-term prognostic relevance of the JAK2V617F mutation were analyzed in 410 Ph-negative MPNs-170 ET, 135 PV, 105 PMF- from two institutions and followed for a mean of 76.7 months (SD 62.1) (mean 87 months (SD 67.8), 70.4 months (SD 56.4), 68 months (SD 57.4), respectively for ET, PV, and PMF). Two hundred and twenty-eight patients were genotyped for JAK2V617F mutation using the JAK2 Ipsogen MutaScreen assay, which involves allele-specific polymerase chain reaction (PCR), and 182 patients were genotyped using melting curve analysis.

**Results:**

In PV patients, JAK2V617F mutation was associated with higher rate in females, lower hemoglobin (Hgb) level, higher leukocyte and platelet count and higher prevalence of thrombosis (p = 0.008, p = 0.018, p = 0.001, p = 0.001, and p = 0.035, respectively). In ET patients, JAK2V617F mutation was associated with higher Hgb and hematocrit (Hct) levels and lower platelet count (p = 0.001, p = 0.001, and p = 0.001, respectively). JAK2V617F-negative ET patients showed a trend towards higher rate of leukemic transformation (p = 0.061). JAK2V617F mutation-positive PMF patients had higher leukocyte count, greater spleen size and showed a trend towards higher Hgb level (p = 0.019, p = 0.042, and p = 0.056, respectively). Among PMF patients with JAK2V617F mutation, the rate of female patients was lower (p = 0.001). Overall survival (OS) in Dynamic International Prognostic Scoring System (DIPSS) - plus high risk PMF patients was shorter compared to the other risk groups (p = 0.001). Leukemia-free survival (LFS) was shorter in DIPSS - plus high risk PMF patients than the other risk groups (p = 0.005). In the entire cohort of Ph-negative MPN patients, JAK2V617F mutation was associated with higher leukocyte count, higher Hgb and Hct levels and lower platelet count, higher frequency of phlebotomies, a trend towards older age, a trend towards greater spleen size, a trend towards a higher prevalence of risk factors for cardiovascular diseases and thrombosis (p = 0.001, p = 0.005, p = 0.001, p = 0.003, p = 0.004, p =0.052, p = 0.056, p = 0.052, and p = 0.059, respectively).

**Conclusion:**

In our study population, it was demonstrated that the presence of JAK2V617F mutation in ET patients was associated with PV-like phenotype. Our study also showed that the presence of the JAK2V617F mutation was associated with increased risk of thrombotic complications. Our results suggest that JAK2V617F mutation is associated with a more pronounced myeloproliferative phenotype in PMF patients. In a large number of Ph-negative MPN patients, our findings support that JAK2V617F mutation is associated with a more aggressive phenotype.

## 1. Introduction

The World Health Organization (WHO) classification system for hematopoietic tumors was last updated in 2016. Myeloproliferative neoplasms (MPNs) are one of the several myeloid malignancies. The Philadelphia-negative (Ph-negative) MPNs are a heterogeneous group of diseases originating at the level of the pluripotent hematopoietic stem cell and include three major diseases: polycthemia vera (PV), essential thrombocythemia (ET) and primary myelofibrosis (PMF) [1]. PV and ET are characterized by clonal erythrocytosis and thrombocytosis, respectively. Other disease features include leukocytosis, splenomegaly, thrombosis, bleeding, microcirculatory symptoms, pruritus and risk of progression to myelofibrosis or acute myeloid leukemia [2]. Characteristic features of PMF are bone marrow reticulin/collagen fibrosis, increased inflammatory cytokine expression, anemia, hepatosplenomegaly, extramedullary hematopoiesis, constitutional symptoms, leukemic progression, and shorter life expectancy [3]. The discovery that almost all PV patients, 55% of ET and 65% of PMF patients express a mutation in the Janus Kinase 2 gene (JAK2V617F) provided a molecular basis for the unregulated hematopoiesis typical of these disorders [2]. Several studies have investigated the impact of JAK2V617F mutation on clinical phenotype in PV, ET, PMF, and entire cohort of Ph-negative MPNs [4–11]. In the same group of patients, the impact of JAK2V617F mutation on overall survival (OS) and leukemia-free survival (LFS) has been the subject of several studies, the results of which were controversial [8,12–17]. This study investigated the clinical and laboratory correlates in 410 patients diagnosed with Ph-negative MPNs - 170 ET, 135 PV, 105 PMF - according to the presence of JAK2V617F mutation. Furthermore, in this large patient series of Ph-negative MPNs with long term follow-up, we evaluated the prognostic relevance of the JAK2V617F mutation.

## 2. Materials and methods

We extracted from our database 410 consecutive Ph-negative MPN patients - 170 ET, 135 PV, 105 PMF - diagnosed between 1995 and 2019. Of the whole study group, 257 and 153 patients were under follow-up at the Division of Hematology of Istanbul University Istanbul Medical Faculty and Hematology Clinic of University of Health Sciences Bakırköy Dr. Sadi Konuk Training and Research Hospital, respectively. All patients fullfilled the 2016 WHO diagnostic criteria for MPNs [18]. Causes of secondary polycythemia, reactive thrombocytosis, familial thrombocytosis, accompanying comorbid disease related anemia, infection related leukocytosis, bone marrow fibrosis other than MF were excluded. Informed consent was obtained from all participants. The study was approved by the local ethics committee and was conducted in accordance with the Declaration of Helsinki. Clinical history, blood count, lactate dehydrogenase (LDH) level, spleen size, presence of cardiovascular risk factors (cigarette, hypertension, diabetes, and dyslipidemia), history of phlebotomy, thrombotic or hemorrhagic complications, death and leukemic transformation were recorded. Dynamic International Prognostic Scoring System (DIPSS)-plus was used for risk stratification in PMF [19]. Unfavorable karyotypes in PMF were described as complex karyotype or one or two abnormalities that include +8, −7/7q−, i(17q), −5/5q−, inv(3), 12p^−^, or 11q23 rearrangement [20]. In 228 of 410 patients, real-time semiquantitative polymerase chain reaction (PCR) with JAK2 MutaScreen assay (Ipsogen, Luminy Biotech, Marseille, France) was used to screen JAK2V617F mutation and the mutant allele burden [21]. In the remaing 182 patients, JAK2V617F mutation was detected by fluorescent resonance energy transfer (FFET) probes and Light Cycler techniques by using Melting Curve analysis [22].

## 3. Statistical analysis

SPSS version 21 (IBM, Armonk, NY, USA) was used for statistical calculations. Numerical variables were summarized by mean (SD). One-sample Kolmogorov–Smirnov test was performed to assess the distribution of the variables in order to use a parametric or nonparametric test. The chi-square statistics were performed to compare categorical variables among the different patient groups categorized according to the JAK2V617F mutational status. The Student t-test and Mann–Whitney U test were used to compare the normally and nonnormally distributed continuous data between two groups, respectively. A p-value of less than 0.05 was regarded as statistically significant; all tests were two-tailed. Seperate OS curves were constructed by Kaplan–Meier method for ET, PV, PMF patients and the whole cohort of Ph-negative MPN patients. Also, Kaplan–Meier estimation was used to plot LFS curves for Ph-negative MPN patients and PMF patients.

## 4. Results

In a total of 410 Ph-negative MPN patients (170 ET, 135 PV, 105 PMF), the frequency of JAK2V617F mutation was 72.7% (n = 298). The frequency of JAK2V617F mutation in ET, PV, and PMF patients was 63.5%, 81.5%, and 76.2%, respectively. In the follow-up of the total cohort of 410 patients, 128 patients (31.2%) underwent phlebotomies. Venous thrombosis was detected in 15% (n = 26), 10% (n = 11) and 15.5% (n = 21) of ET, PV and PMF patients, respectively. Of the 26 ET patients with venous thrombosis, 12 presented with abdominal vein thrombosis, 7 with cerebral vein thrombosis, 4 with deep vein thrombosis (DVT), 2 with pulmonary embolism (PE) and 1 patient presented with concomitant DVT and abdominal vein thrombosis. Of the 11 PMF patients with venous thrombosis, 9 presented with abdominal vein thrombosis,1 with DVT and 1 patient presented with PE. Of the 21 PV patients with venous thrombosis, 11 presented with abdominal vein thrombosis, 6 with DVT, 2 with cerebral vein thrombosis, 1 with PE and 1 patient presented with concomitant DVT and cerebral vein thrombosis. Arterial thrombosis was detected in 22% (n = 37), 12% (n = 13) and 24% (n = 32) of ET, PV, and PMF patients, respectively. Of the 37 ET patients with arterial thrombosis, 13 presented with coronary artery thrombosis, 12 with cerebral artery thrombosis, 5 with peripheral artery thrombosis, 4 with concomitant coronary and cerebral artery thrombosis, 1 with concomitant renal and coronary artery thrombosis, 1 with concomitant cerebral and peripheral artery thrombosis and 1 patient presented with concomitant coronary and peripheral artery thrombosis. Of the 13 PMF patients with arterial thrombosis, 7 presented with coronary artery thrombosis, 3 with peripheral artery thrombosis, 2 with concomitant coronary and cerebral artery thrombosis and 1 patient presented with cerebral artery thrombosis. Of the 32 PV patients with arterial thrombosis, 18 presented with coronary artery thrombosis, 11 with cerebral artery thrombosis, 1 with peripheral artery thrombosis, 1 with concomitant coronary and cerebral artery thrombosis and 1 patient presented with concomitant cerebral and renal artery thrombosis. Bleeding was observed in 12.7% (n = 52) of the whole study population. The source of bleeding was gastrointestinal tract, oral mucosa, cutaneous, intracranial, alveolar, ocular, concomitant cutaneous, oral mucosa, and gastrointestinal tract in 5.6% (n = 23), 3.2% (n = 13), 2%(n = 8), 1% (n = 4), 0.5% (n = 2), 0.2% (n = 1), 0.2% (n = 1), respectively of the 52 bleeding patients. Of the 17 ET patients with bleeding, the source of bleeding was gastrointestinal tract, oral mucosa, cutaneous, ocular and intracranial in 8, 4, 3, 1, and 1 patient, respectively. Of the 21 PMF patients with bleeding, the source of bleeding was gastrointestinal tract, oral mucosa, cutaneous, intracranial, concomitant cutaneous, oral mucosa and gastrointestinal tract and alveolar in 9, 6, 2, 2, 1, and 1 patient, respectively.The cause of mortality in PV patients were sudden cardiac death (SCD) (n = 8) and leukemic transformation (n = 2). In ET patients, the cause of mortality was respiratory failure (n = 3), liver failure (n = 1), SCD (n = 12) and leukemic transformation (n = 2). In PMF patients, the cause of mortality was respiratory failure (n = 9), SCD (n = 18) and leukemic transformation (n = 8).

### 4.1. Comparison of Ph-negative myeloproliferative neoplasms stratified by the JAK2V617F mutation

Total cohort in our study included 170 ET, 135 PV, and 105 PMF patients (410 Ph-negative MPNs). Mean duration of follow-up was 76.7 months (SD 62.1). The mean age of the total cohort (50% females) at diagnosis and at time of data collection was 53.3 (SD 14.9) and 60.3 years (SD 14.8), respectively.

Clinical and laboratory characteristics of Ph-negative MPN patients stratified by the JAK2V617F mutation are outlined in Table 1.

Sex at the time of data collection showed no difference between JAK2V617F-positive and -negative Ph-negative MPN subgroups. There was a trend towards older age at diagnosis in JAK2V617F-mutated MPN patients compared to JAK2V617F-unmutated patients (mean 54.34 [15] and 51.39 [14.79], respectively; p = 0.052). Ph-negative MPN patients with JAK2V617F mutation presented with higher leukocyte count, higher Hgb and Hct levels and lower platelet count at diagnosis compared to patients without the mutation (p = 0.001, p = 0.005, p = 0.001, and p = 0.003, respectively). LDH levels were similar for the groups.

There was a trend towards greater spleen size in JAK2V617-positive MPN patients compared to JAK2V617F-negative patients (mean 148.33 mm [43.08] and 137.74 mm [31.87], respectively; p = 0.056).

JAK2V617F positive MPN patients showed a trend towards higher prevalence of risk factors for cardiovascular diseases and thrombosis compared to JAK2V617F-negative patients (74.8% and 65.2%, respectively; p = 0.052 and 33.6% and 25.9%, respectively; p = 0.059). The frequency of phlebotomy in JAK2V617F-mutated MPN patients was higher than JAK2V617F-unmutated patients (35.2% and 20.5%, respectively; p = 0.004). JAK2V617F-mutated MPN showed a higher yet not statistically significant rate of bleeding events than the JAK2V617F-negative group (14.4% and 8%, respectively; p = 0.083).

Duration of follow-up in patients with and without JAK2V617F mutation were 82.04 months (SD 63.07) and 74.67 months (SD 61.73), respectively (p = 0.224). The rate of death and leukemic transformation did not differ between the two groups.

#### 4.1.1. Survival curves: Kaplan–Meier survival curves in patients with Ph-negative myeloproliferative neoplasms according to JAK2V617F mutation

**Figure 1:**
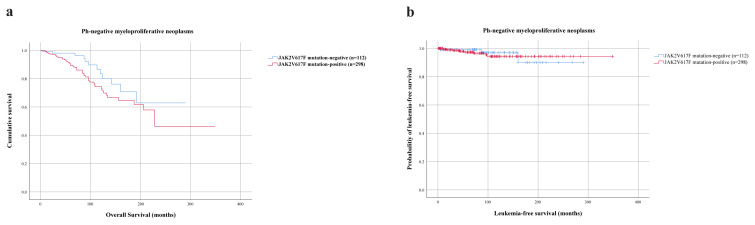
Survival outcomes and Leukemia-free survival in Ph-negative myeloproliferative neoplasms (n = 410). a. Survival analysis of patients diagnosed with Ph-negative myeloproliferative neoplasms according to JAK2V617F mutation. OS was similar for JAK2V617F mutation positive and JAK2V617F mutation negative patients (p = 0.199). b. Kaplan–Meier plot showing LFS in patients diagnosed with Ph-negative myeloproliferative neoplasms according to JAK2V617F mutation. LFS showed no difference with respect to JAK2V617F mutation (p = 0.748).

JAK2V617F mutation was tested in survival analysis for influence on OS and LFS in patients diagnosed with Ph-negative MPNs. Kaplan–Meier plots revealed similar OS for JAK2617F mutated (n = 298) and JAK2617F-unmutated (n = 112) patients (mean 234 months; 95% CI: 204–264 and 230 months; 95% CI: 199–260, respectively; p = 0.199) (Figure 1a). Moreover, comparison across patients with JAK2V617F mutation-positive and JAK2V617F mutation-negative patients showed no significant difference in LFS (mean 338 months; 95% CI: 328–349 and 275 months; 95% CI: 257–293, respectively; p = 0.748) (Figure 1b).

### 4.2. Comparison of essential thrombocythemia patients according to JAK2V617F mutation

One hundred and eight of 170 ET patients harbored JAK2V617F mutation (63.5). The mean duration of follow-up was 87 months (SD 67.8). The mean age of ET patients (60% females) at diagnosis and at time of data collection was 50.2 (SD 15.4) and 57.9 years (SD 15.6), respectively.

Clinical and laboratory features of ET patients according to JAK2V617F mutation are summarized in Table 2.

JAK2V617F-mutated ET patients showed a higher yet not statistically significant rate of females compared to JAK2V617F-unmutated patients (64.8% and 51.6%, respectively; p = 0.091). Age at diagnosis and age at time of data collection were similar between JAK2V617F-mutated and -unmutated ET patients. JAK2V617F-mutated ET patients showed higher Hgb and Hct levels and lower platelet count at diagnosis compared to JAK2V617F-unmutated patients (p = 0.001, p = 0.001, and p = 0.001, respectively). Leukocyte count, LDH level, and spleen size at diagnosis were not different between the groups.

Prevalence of risk factors for cardiovascular diseases, rate of bleeding and thrombosis were similar between the two groups.

Duration of follow-up for JAK2V617F-mutated and -unmutated patients was 85.23 months (SD 67.87) and 90.1 months (SD 68.18), respectively (p = 0.560). There was a trend towards higher rate of leukemic transformation in the JAK2V617F-unmutated group compared to the JAK2V617F-mutated group (3.2% and 0, respectively; p = 0.061). The rate of death showed no difference between the groups.

#### 4.2.1. Survival curves: Kaplan–Meier plots in patients with essential thrombocythemia according to JAK2V617F mutation

**Figure 2:**
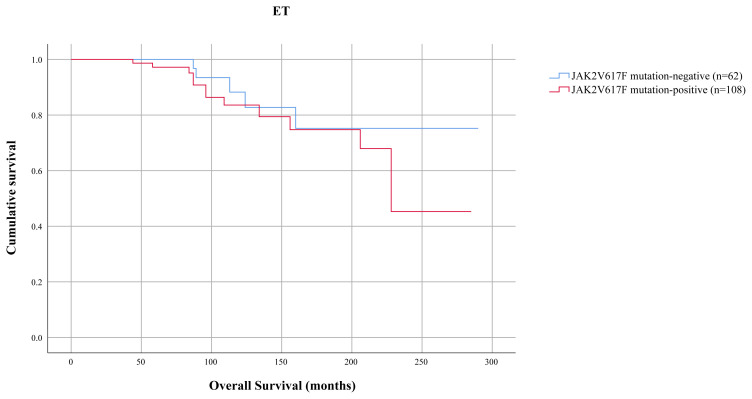
Kaplan–Meier estimate of survival in ET patients according to JAK2V617F mutation. OS was similar for JAK2V617F mutation positive and negative ET patients (p = 0.879)

JAK2V617F mutation was tested in survival analysis for influence on OS in patients diagnosed with ET. JAK2V617F-mutated and unmutated ET patients showed no significant difference in OS (mean 237 months; 95% CI: 208–267 and 251 months; 95% CI:221–282, respectively; p = 0.879) (Figure 2).

### 4.3. Comparison of *polycythemia vera* patients according to JAK2V617F mutation

Among the 135 patients diagnosed with PV, the frequency of JAK2V617F mutation was 81.5% (n = 110). The mean duration of follow-up was 70.4 months (SD 56.4). The mean age of PV patients (65.2% males) at diagnosis and at time of data collection was 55.01 (SD 14.1) and 61.2 years (SD 14.1), respectively.

Clinical and laboratory characteristics of PV patients stratified by JAK2V617F mutation are outlined in Table 3.

The rate of female PV patients was higher in the JAK2V617F-positive group compared to the JAK2V617F-negative group (40% and 12%, respectively; p = 0.008). Compared to JAK2V617F mutation-negative PV patients, JAK2V617F mutation-positive PV patients showed a higher yet not statistically significantmean age at diagnosis and age at time of data collection (56.04 (SD 13.90) and 50.52 (SD 14.15), respectively; p = 0.082 and 62.25 (SD 13.91) and56.68 (SD 14.51), respectively; p = 0.071).

JAK2V617F-mutated PV patients showed lower Hgb levels, higher leukocyte and platelet counts at diagnosis compared to JAK2V617F-unmutated patients (p = 0.018; p = 0.001 and p = 0.001, respectively). Hct and LDH levels and spleen size at diagnosis were not different between the groups. JAK2V617F-mutated PV patients showed higher prevalence of thrombosis compared to JAK2V617F-unmutated patients (42.7% and 20%, respectively; p = 0.035)

Prevalence of risk factors for cardiovascular diseases, rates of phlebotomy, and bleeding were similar between the two groups.

Duration of follow-up in patients with and without JAK2V617F mutation were 70.81 months (SD 56.76) and 68.7 months (SD 55.86), respectively (p = 0.883). At the end of data collection period, the rates of death and leukemic transformation did not differ between the two groups.

#### 4.3.1. Survival curves: Kaplan–Meier plots in patients with *polycythemia vera* according to JAK2V617F mutation

**Figure 3:**
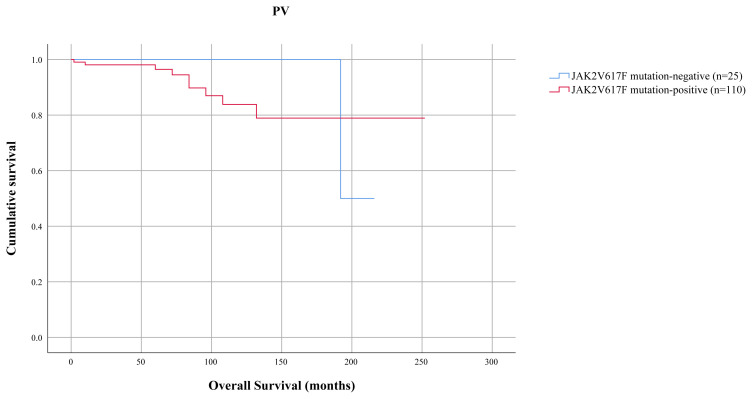
Overall survival comparison among 135 patients with PV divided by JAK2V617F mutation. OS was similar between JAK2V617F mutation-positive and JAK2V617F mutation-negative PV (p = 0.887).

In PV patients, JAK2V617F mutation was tested in univariate survival analysis for influence on OS. OS did not differ between JAK2V617F-mutated and JAK2V617F-unmutated PV patients (mean 217 months; 95% CI: 196–238 and 215 months; 95% CI: 213–217, respectively; p = 0.887) (Figure 3).

### 4.4. Comparison of *primary myelofibrosis* patients according to JAK2V617F mutation (n = 105)

For the 105 PMF patients included in the study, the frequency of JAK2V617F mutation was 76.2% (n = 80). Mean duration of follow-up was 68 months (SD 57.4). The mean age of PMF patients (46.7% males) at diagnosis and at time of data collection was 56.93 (SD 14.37) and 63.09 years (SD 13.64), respectively. According to DIPSS-plus risk stratification, PMF patients were divided into low, intermediate-1, intermediate-2 and high risk groups (19% (n = 20), 37% (n = 39), 33% (n = 35) and 11% (n = 11).

Clinical and laboratory characteristics of PMF patients according to JAK2V617F mutation are summarized in Table 4.

The rate of females was higher in the JAK2V617F-unmutated PMF patients compared to the JAK2V617F-mutated patients (84% and 43.8%, respectively; p = 0.001). Mean age at diagnosis and age at time of data collection were not different between PMF patients with and without JAK2V617F mutation.

JAK2V617F-mutated PMF patients showed higher leukocyte count at diagnosis compared to JAK2V617F-unmutated PMF patients (p = 0.019). In JAK2V617F- mutated PMF patients, a trend towards higher Hgb level at diagnosis was observed compared to JAK2V617F-unmutated PMF patients (p = 0.056). Hct and LDH levels and platelet count at diagnosis did not differ between the groups. JAK2V617F- mutated PMF patients showed greater spleen size compared to JAK2V617F-unmutated patients (mean 193 mm (SD 48) and 171 mm (SD 40), respectively; p = 0.042)

JAK2V617F-mutated PMF patients showed a higher yet not statistically significant prevalence for risk of cardiovascular diseases and rate of bleeding compared to patients without mutation (71.3% and 52%, respectively; p = 0.075 and 23.8% and8%, respectively; p = 0.086). The frequency of thrombosis was similar between JAK2V617F-mutated and -unmutated patients.

Duration of follow-up in patients with and without JAK2V617F mutation were 65.74 months (SD 58.19) and 75.36 months (SD 55.32), respectively (p = 0.304). At the end of data collection period, the rate of death and leukemic transformation were similar between the two groups.

#### 4.4.1. Survival curves: Kaplan-Meier plots in patients with *Primary myelofibrosis* according to JAK2V617F mutation and DIPSS-plus risk stratification

**Figure 4:**
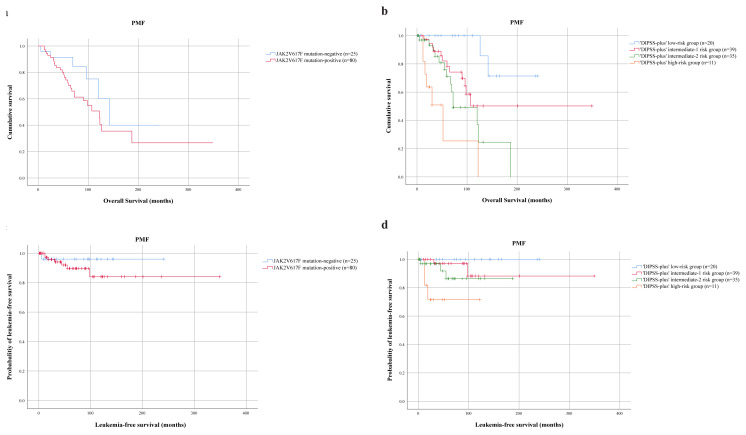
Survival outcomes and leukemia-free survival in primary myelofibrosis patients (n = 105). a. Kaplan–Meier plot showing OS in PMF patients according to JAK2V617F mutation. OS was similar between JAK2V617F-positive and JAK2V617F-negative patients (p = 0.134). b. Survival analysis of patients diagnosed with PMF according to DIPSS-plus risk stratification. OS was shorter in DIPSS-plus high risk PMF patients with respect to other risk groups (p = 0.001). c. LFS comparison between 80 JAK2V617F-mutated and 25 JAK2V617F-unmutated patients with PMF. LFS was similar between JAK2V617F-mutated and -unmutated PMF patients (mean 309 months; 95% CI: 274–343 and 239 months; 95% CI:221–258, respectively; p = 0.354). d. LFS data of PMF patients according to DIPSS-plus risk stratification. PMF patients with DIPSS-plus high risk group had significantly shorter LFS compared to other risk groups **(**p = 0.005).

For PMF patients, JAK2V617F mutation and DIPSS-plus risk stratification were tested in univariate survival analysis for influence on OS and LFS. JAK2V617F positive (n = 80) and JAK2V617F-negative PMF (n = 25) patients showed no significant difference in OS (mean 162 months; 95% CI: 114–210 and 161 months; 95% CI: 110–212, respectively; p = 0.134) (Figure 4a). Comparison across DIPSS-plus high risk patients (n = 11), intermediate-2 (n = 35), intermediate-1 (n = 39), and low risk patients (n = 20) demonstrated that DIPSS-plus high risk patients lived shorter compared to the other risk groups (p = 0.001) (mean 59 months; 95% CI: 14–104, 104 months; 95% CI: 75–133, 222 months; 95% CI: 163–281 and 216 months; 95% CI: 177–254 respectively; p = 0.001) (Figure 4b). LFS was not different between JAK2V617F-mutated and -unmutated PMF patients (mean 309 months; 95% CI: 274–343 and 239 months; 95% CI: 221–258, respectively; p = 0.354) (Figure 4c). LFS was shorter in DIPSS-plus high risk PMF patients compared to the other risk groups (mean 109 months; 95% CI: 73–145 in high risk, 167 months; 95% CI: 146–188 in intermediate-2, 327 months; 95% CI: 289–365 in intermediate-1 and 236 months; 95% CI: 213–260 in low risk, respectively; p = 0.005) (Figure 4d).

## 5. Discussion

Our study included a large number of patients diagnosed with Ph-negative MPNs (n = 410) with a long duration of follow-up- 170 ET patients with a mean follow-up duration of 87 months (SD 67.8), 135 PV patients with a mean follow-up duration of 70.4 months (SD 56.4) and 105 PMF patients with a mean follow-up duration of 68 months (SD 57.4). In a 2021 update of Ph-negative MPNs, it was reported that JAK2V617F mutation was displayed in 96%, 55%, and 65% of PV, ET, and PMF patients, respectively [2]. However, in a systematic review, the frequency of the JAK2V617F mutation in Ph-negative MPNs showed marked variation with incidence rates ranging between 46.7% and 100% in PV, 31.3% and 72.1% in ET, and 25%–85.7% in PMF [23]. Differences in the literature may be attributed to the heterogeneous diagnostic techniques [23]. In our study, we used a semiquantitative assay -JAK2 MutaScreen- with a sensitivity of 2% in 228 patients and real-time PCR assay using FFET probes and melting curve analysis with a sensitivity of 10% in the remaining 182 patients [21,22]. In our study, the frequency of JAK2V617F mutation in PV was higher compared to ET and PMF patients (81.5%, 63.5% and 76.2%, respectively).

Several studies have reported the comparison of ET patients according to JAK2V617F mutation [4,5,9,12,13,24–36]. In a study including 218 consecutive ET patients, the presence of JAK2V617F mutation retained a negative prognostic impact for predicting thrombosis [4]. In another study including 53 ET patients, JAK2V617F mutation showed significant correlation with higher leukocyte counts, higher Hgb levels and thrombotic events while age, sex, platelet count, frequency of splenomegaly, and bleeding events did not differ between the JAK2617F-positive and JAK2V617F-negative ET patients [24]. In a study including 102 ET patients, females were reported to more frequently harbor the JAK2V617F mutation and JAK2V617F mutated patients were found to be older and had higher leucocyte counts [25]. Another study including 150 ET patients showed that JAKV617F mutated subgroup was associated with advanced age, higher Hgb level and leukocyte counts [12]. In the same study, sex, platelet count, palpable splenomegaly, thrombosis, and hemorhage at presentation and at follow-up showed no difference according to JAK2V61F mutation [12]. In a metaanalysis, however, it was reported that JAK2V617F increased the risk of arterial and venous thrombosis in ET [5]. Another metaanalysis including 325 published articles also supported that JAK2V617F positive ET was associated with increasing odds of thrombosis [26]. In a comprehensive study including 395 ET patients, JAK2V617F-positive patients had significantly higher Hgb level and leukocyte counts but lower platelet counts compared to JAK2V617F-negative patients [27]. In a study including 275 ET patients, JAK2V617F-positive patients were older and displayed higher Hgb and Hct levels and higher incidence of splenomegaly, but lower platelet count and lower incidence of hemorrhagic events compared with patients without the mutation while sex, leukocyte count, incidence of thrombosis was comparable between the groups [30]. In a study, JAK2V617F-positive ET patients displayed higher Hgb and Hct levels whereas leukocyte and platelet counts, sex, age, disease duration did not differ between JAK2V617F-positive and negative ET patients [31]. In another study including 92 ET patients, significantly higher values were also found for Hgb level and leukocyte count in the JAK2V617F mutation positive group yet platelet count, LDH level, age, sex and thrombosis did not differ between JAK2V617F-positive and negative patients [32]. In another study, it was demonstrated that JAK2V617F-positive ET patients showed advanced age, higher leukocyte count and Hgb level whereas platelet count was similar between JAK2V617F-positive and -negative groups [33]. In a study including 111 ET patients, the presence of JAK2V617F mutation correlated with older age, higher levels of Hgb and Htc levels, greater probability of having splenomegaly at diagnosis while mutation positive and negative groups showed no difference with respect to sex, probability of hemorrhagic events, leukocyte count, and LDH levels [13]. In a very large ET series including 806 patients, JAK2V617F-positive patients had significantly increased Hgb level, neutrophil count, more venous thrombosis compared to JAK2V617F-negative ET patients [36]. In a previous study including 107 ET patients, JAK2V617F-positive patients presented with higher Hgb and Hct levels and lower platelet count and more prevalent splenomegaly while age, sex, leukocyte count, LDH level, spleen size, rate of bleeding complications and thrombosis and duration of follow-up did not differ between the groups [9]. In our study, JAK2V617F-positive ET patients displayed higher Hgb and Hct levels and lower platelet count at diagnosis compared to JAK2V617F-negative patients while no differences were observed between the groups regarding age, sex, leukocyte count, LDH level, spleen size at diagnosis, duration of follow-up, prevalence of risk factors for cardiovascular diseases, rate of bleeding and thrombosis. In our study, JAK2V617F-positive ET patients showed higher Hgb and Hct levels in line with previous studies yet as opposed to the study by Wong et al. [9, 12, 13, 24, 25, 27–36]. In agreement with some previous studies but in contrast to the study by Pósfai et al., in which JAK2V617F mutation was associated with increased platelet count and in contrast to some studies in which the platelet count was reported not to differ between JAK2V617F-positive and negative patients, our study demonstrated that the platelet count was lower in in JAK2V617F-positive ET patients patients [9,12,24,25,27–34]. Contrary to most previous studies showing association between the JAK2V617F mutation and higher leukocyte count yet confirming some of the previous reports, our JAK2V617F-positive and negative ET patients showed no difference with respect to leukocyte counts [9,12,13,24,25,27–36]. Confirming previous data, we found no difference in LDH levels between JAK2V61F-positive and -negative ET patients [9,29,32,34]. Some previous studies showed an association between JAK2V617F mutation and older age in ET patients while others found no association [9,12,13,24,25,29–34]. In line with the aferomentioned several studies, age was similar between our JAK2V617F-positive and -negative ET patients [9,24,29,31,32,34]. Consistent with most previous observations but contrary to the study by Wong GC et al., sex did not differ according to JAK2V617F mutation in our ET patients [9,12,13,24,25,29,30,32,34]. Contrary to previous some reports but in line with other observations, we found no association between JAK2V617F mutation and spleen size [9,12,13,24,29,30,34]. In line with previous studies, the duration of follow-up between our ET patients with and without the JAK2V617F mutation was similar [9,12,25,31]. Consistent with previous data yet contrary to the observation by Palandri et al., we found no difference in the rate of bleeding between JAK2V617F-positive and JAK2V617F-negative patients [9,12,13,24,29,30]. Consistent with previous observations but contrary to some others, the rate of thrombosis was similar between our JAK2V617F-positive and -negative ET patients [4,5,9,12,24,26,28–30,32,34,36]. Finally, similar to most of the aferomentioned studies, our ET patients had some hematological features resembling PV with significantly increased Hgb and Hct levels yet contrary to some of the previous studies, our ET patients did not promote a PV phenotype in terms of vascular events [4,5,12,13,24,26–36].

Because ~95% of PV patients harbor JAK2V617F mutations, limited studies have previously compared JAK2V617F-positive and JAK2V617F-negative PV patients [6,7,13,14,32,34,37–39]. In one study including 108 PV patients, patients with JAK2V617F mutation had higher platelet, leukocyte counts, and LDH levels and were older compared to JAK2V617F-unmutated patients while the JAK2V617F-mutated and -unmutated PV patients showed no difference for Hgb, sex, and thrombosis [32]. In a study including 80 PV patients, PV patients carrying the JAK2V617F mutation had higher leukocyte and platelet counts and were more prone to have splenomegaly compared to patients without the mutation [34]. In the same study, median age, sex, Hgb, and LDH levels, frequency of thrombosis did not differ between JAK2V617F-positive and JAK2V617F-negative PV patients [34]. Vannucchi et al. reported that in PV patients, JAK2V617F mutation clusters with older age, higher Hgb level, leukocytosis, and lower platelet count [37]. In a study including 92 PV patients, JAK2V617F-mutated patients were associated with splenomegaly and they had higher leukocyte and platelet counts and showed a significant increase in LDH levels compared to JAK2V617F-unmutated patients [14]. In the same study, the rate of males was higher in JAK2V617F-negative PV patients compared to JAK2V617F-positive patients while Hgb level did not differ between patients with and without the mutation [14]. In a study including 83 PV patients, leukocyte and platelet counts and Hct level were similar between JAK2V617F-mutated and -unmutated patients [39]. In a series of 43 PV patients, age, sex, presence of splenomegaly and thrombosis, Hgb and Hct levels, platelet count, and LDH level were not different between JAK2V617F-mutated and unmutated patients while -mutated patients had higher leukocyte count and longer duration of follow-up [13]. In our study including 135 PV patients, the rate of females was higher in JAK2V617F-positive patients and JAK2V617F-positive patients showed lower Hgb level, higher leukocyte and platelet counts at diagnosis and a higher prevalence of thrombosis while no differences were observed for duration of follow-up, prevalence of cardiovascular risk factors, rates of phlebotomy and bleeding, Hct, and LDH levels and spleen size at diagnosis between the groups. Moreover, our JAK2V617F-positive PV patients displayed a higher yet not statistically significant mean age at diagnosis and age at time of data collection compared to JAK2V617F-negative patients. In our study, PV patients with JAK2V617F mutation displayed lower Hgb level compared to JAK2V617F-unmutated PV patients in contrast with some studies showing correlation between JAK2V617F mutation and higher Hgb level and other studies showing no correlation between JAK2V617F mutation and Hgb level [13,14,32,34,37,38]. In line with previous observations, our JAK2V617F-mutated and -unmutated PV patients showed similar Hct levels [13,39]. Consistent with previous reports yet in contrast to the study by Ibrahim et al., our JAK2V617F-mutated PV patients displayed higher leukocyte counts compared to patients without the mutation [16,13,14,32,34,37–39]. In our study, JAK2V617F-mutated PV patients showed higher platelet counts in agreement with most of the previous data yet in contrast with some studies [6,7,13,14,32,34,37,39]. Confirming some previous studies but at variance with some others, we found no correlation between JAK2V617F mutation and LDH level [7,13,14,32,34]. In line with some studies yet opposed to the others showing correlation between JAK2V617F mutation and older age, our PV patients showed no difference for age with respect to the presence of JAK2V617F mutation [13,32,34,37]. In contrast with most of the previous studies yet in line with the study by Soliman et al., the rate of female patients was higher in our PV patients with JAK2V617F mutation [13,14,32,34]. In our PV patients, spleen size was similar between JAK2V617F-mutated and -unmutated patients as opposed to most of the studies yet in line with the study by Speletas M et al. [7,13,14,34]. In our PV patients, duration of follow-up was similar between JAK2V617F-mutated and -unmutated patients as opposed to the finding by Speletas et al [13]. In our study, prevalance of thrombosis was higher in JAK2V617F-mutated PV patients compared to JAK2V617F-unmutated patients as opposed to the previous data [13,32,34]. To our knowledge, there is no previous data regarding the prevalence of risk factors for cardiovascular diseases, rates of phlebotomy and bleeding in PV patients according to JAK2V617F mutation. In our study, we observed no correlation with the aforementioned parameters and JAK2V617F mutation. Reported data regarding the correlation of JAK2V617F mutation with advanced age, decreased platelet counts, thrombotic risk, splenomegaly have yielded contradictory results [40]. In our large number of PV patients, we demonstrated that the presence of the JAK2V617F mutation promoted a distinct phenotype characterized by female predominance, lower Hgb level, higher leukocyte and platelet counts and higher prevalence of thrombosis.

Several studies have investigated the clinical correlations of JAK2V617F mutation in patients with PMF [8,9,15,17,41–44]. In a study including 152 PMF patients, JAK2V617F-mutated patients had higher leukocye count compared to JAK2V617F-unmutated patients while age, sex, Hgb level, LDH level, platelet count and spleen size were not different between patients with and without the mutation [15]. In a series of 117 PMF patients from a single center, Tefferi et al. reported no significant impact of the presence of JAK2V617F mutation on sex, Hgb level, leukocyte count, platelet count, LDH level, spleen size, bleeding history, but the presence of the JAK2V617F mutation was found to be associated with older age and history of thrombosis [8]. In another study including 304 PMF patients, the presence of JAKV617F mutation contributed to laboratory and clinical abnormalities including higher Hgb level and leukocyte count and development of marked splenomegaly [17]. In another study including 199 PMF patients, the presence of JAK2V617F mutation showed no correlation with sex, Hgb level, platelet count, and incidence of thrombosis while JAK2V617F-mutated patients were significantly of older age and showed a trend towards higher leukocyte count [41]. In a study including 186 PMF patients, JAK2V617F-mutated patients had significantly higher Hgb level, leukocyte count and platelet count compared to JAK2V617F-unmutated patients [42]. In the aferomentioned study, there was no impact of the mutated genotype on age, sex, LDH level, duration of follow-up and the presence of a palpable spleen greater than 15 cm [42]. In a series of 77 Turkish PMF patients, it was demonstrated that JAK2V617F-mutated patients presented with significantly higher leukocyte count, Hb and Hct levels and included a lower rate of female patients compared to JAK2V617F-unmutated patients while no significant difference was reported for age, platelet count, LDH level, spleen size, duration of follow-up and prevalance of thrombosis [9]. In the present study including 105 PMF patients, JAK2V617F-mutated PMF patients included a lower rate of female patients and they displayed higher leukocyte count, greater spleen size, a trend towards higher Hgb level. In our study, age, Hct, and LDH levels, platelet count, duration of follow-up and prevalence of thrombosis were found to be similar between JAK2V617F-mutated and -unmutated patients. Moreover, our JAK2V617F-mutated PMF patients showed a higher yet not statistically significant prevalence of cardiovascular risk factors and rate of bleeding compared to -unmutated patients. In our study, JAK2V617F-mutated PMF displayed a trend towards higher Hgb level in line with some reports yet as opposed to other data [8,9,15,17,41–44]. In contrast with one previous study, our JAK2V617F-mutated and -unmutated PMF patients showed no difference in Hct levels [9]. In agreement with most studies yet opposed to some others, leukocyte count in our JAK2V617F-mutated PMF patients was higher [8,9,15,17, 41–43]. Confirming most previous observations but contrary to some others, we found no correlation between JAK2V617F mutation and platelet count [8,9,15,17,41–43]. In accordance with previous observations but in contrast with the study by Helbig et al., our JAK2V617F-mutated and -unmutated PMF patients showed no difference in LDH level [8,9,15,41,42]. In line with some previous studies yet as opposed to some others, we did not observe any impact of JAK2V617F mutation on age [8,9,15,17,41–43]. In contrast with some reports yet confirming the findings of a previous study, JAK2V617F-mutated PMF patients included a lower rate of females [8,9,15,17,41]. Contrary to previous studies yet in line with the report by Barosi et al., our JAK2V617F-mutated PMF patients displayed greater spleen size compared to JAK2V617F-unmutated patients [8,9,15,17,41–43]. Consistent with previous reports, duration of follow-up of our JAK2V617F-mutated and -unmutated PMF patients was similar [9,41]. In agreement with previous studies but in contrary to the study by Tefferi et al., we found no difference in the prevalance of thrombosis [8,9,17]. Rate of bleeding was similar between our PMF patients with and without the JAK2V617F mutation, consistent with the observation by Tefferi et al. [8]. As a whole, we found a significant association between the presence of JAK2V617F mutation and a more marked myeloproliferative phenotype in PMF patients.

Several studies have reported the comparison of an entire cohort of Ph-negative MPNs according to the JAK2V617F mutation [10, 11, 13, 45, 46]. In a study including a total cohort of 186 patients diagnosed with MPN, individuals harboring the JAK2V617F mutation were reported to have higher risk for VTE but not for arterial thrombosis or bleeding complications [10]. In another study including 166 MPN patients, the presence of the JAK2V617F mutation correlated with older age, higher levels of Hct and Hgb while sex, platelet count, LDH level, rate of thrombosis and bleeding complications, follow-up duration did not differ between patients with and without the mutation [13]. In a series of 412 MPN patients, there was a correlation between the JAK2V617F mutation and advanced age, higher leukocyte count and Hgb level and presence of thrombosis [11]. In a study including 88 MPN patients, the risk of thrombosis and bleeding were not affected by the presence of the JAK2V617F mutation [45]. In a series of 148 MPN patients (including PV and ET), JAK2V617F-mutated patients displayed older age, higher Hgb level and leukocyte count, lower platelet count and more prevelant splenomegaly compared to JAK2V617F-unmutated patients while sex was not different between the groups [46]. In our study including 410 MPN patients (170 ET, 135 PV, 105 PMF), JAK2V617F-mutated patients displayed a trend towards older age at diagnosis, higher leukocyte count, higher Hgb and Hct levels and lower platelet count, a trend towards greater spleen size, higher frequency of phlebotomy, a trend towards higher prevalence of cardiovascular risk factors and higher rate of thrombosis while sex, LDH level, rate of bleeding events, duration of follow-up did not differ between patients with and without the mutation. In our study, JAK2V617F-mutated MPN patients displayed higher Hgb level in agreement with previous reports [11,13, 45, 46]. Moreover, consistent with the report of Speletas et al., we found a correlation between the JAK2V617F mutation and higher Hct level [13]. Confirming previous data, JAK2V617F-mutated MPN patients showed higher leukocyte counts compared to JAK2V617F-unmutated patients [11,45,46]. In accordance with the previous observation of Karkucak et al. but in contrast to the study by Lieu et al., our JAK2V67F-positive MPN patients had lower platelet counts [45,46]. Consistent with the report of Speletas et al., LDH level did not differ between our MPN patients with and without JAK2V617F mutation [13]. In our study, there was a trend towards older age in JAK2V617F-mutated MPN in line with previous reports yet as opposed to the finding by Lieu et al. [11,13,45,46]. Confirming previous reports, we found no sex difference in our MPN patients according to the JAK2V617F mutation [13,45,46]. Consistent with previous reports showing association between the JAK2V617F mutation and splenomegaly in MPN, our JAK2V617F-mutated MPN patients also showed a trend towards greater spleen size [45,46]. In line with the report by Speletas et al. yet contrary to the report by Lieu et al., duration of follow-up was similar between our MPN patients with and without the JAK2V617F mutation [13,45]. In line with a study including a large series of MPN patients, which showed correlation between the JAK2V617F mutation and thrombosis and with the study by Borowczyk et al., which showed higher incidence of VTE in patients with the JAK2V617F mutation, we found a trend towards a higher prevalence of thrombosis in our JAK2V617F-mutated MPN patients [10,11]. In contrast with the aforementioned findings, some other previous studies found no significant increased risk of thrombosis in JAK2V617F-mutated patients [13,45]. Confirming previous data, the rate of bleeding events was similar between our MPN patients with and without JAK2V617F mutation [10,13,45]. Consequently, in our MPN patients, the presence of the JAK2V617F mutation promoted a PV phenotype characterized by older age, higher leukocyte count, higher Hgb and Hct levels, lower platelet count, greater spleen size, thrombotic risk and higher rate of phlebotomy.

There are several reports that have highlighted the impact of JAK2V61F mutation on outcomes of PV, ET, PMF and the entire cohort of Ph-negative MPN patients [8, 9, 12–14,16, 17, 19, 30, 41, 43, 47, 48]. To our knowledge, there is limited data regarding the outcome of PV patients according to the JAK2V617F mutation. In a study including 60 PV patients, the rate of leukemic transformation showed no difference according to the presence of the JAK2V617F mutation [47]. Similarly, in our study which includes 135 PV patients, the rate of leukemic transformation was not different between JAK2V617F-positive and JAK2V617F-negative group [47]. Moreover, death and OS were similar between our JAKV617F-mutated and JAK2V617F-unmutated PV patients. In a study including 107 ET patients, OS was similar between JAK2V617F-positive and JAK2V617F-negative patients [48]. In another study including 141 ET patients, the 10-year OS was not different in patients with and without the JAK2V617F mutation. Moreover, in that study, the rate of death and blastic transformation were not different in patients with and without the mutation [16]. In line with the aforementioned observation, another study reported that no difference was observed in the rates of death with respect to JAK2V617F mutational status in ET patients [9]. In contrast with the previous data, another study including 111 ET patients reported that patients carrying the JAK2V617F mutation had a three-fold higher probability of death compared to those without the JAK2V617F mutation [13]. In a study including 150 ET patients, the number of deaths was significantly higher in JAK2V617F-mutated patients compared to JAK2V617F-unmutated patients [12]. In the same study, multivariate analysis did not show the presence of JAK2V617F mutation as a significant predictor in ET patients for OS [12]. On the contrary, in one study which evaluated the correlation between the JAK2V617F mutation and OS, it was demonstrated that JAK2V617F-mutated ET patients had shorter OS [14]. In one study including 275 ET patients, the incidence of disease transformation was not different between JAK2V617F-positive and JAK2V617F-negative patients [30]. In our study, there was a trend towards higher rate of leukemic transformation in JAK2V617F-unmutated ET patients compared to mutated patients while the rate of death was not different between the groups. Moreover, JAK2V617F-mutated and -unmutated ET patients showed no significant difference in OS. In a previous study including 77 PMF patients, the rates of leukemic transformation and death were similar between the JAK2V617F-positive and -negative groups [9]. In another study including PMF patients, the presence of JAK2V617F mutation had no impact on OS and LFS [48]. On the contrary, in a series of 152 PMF patients, JAK2V617F-positive patients showed significantly worse survival compared to JAK2V617F-negative patients [15]. In a series of 117 PMF patients, JAK2V617F mutation showed no significant impact on either survival or leukemic transformation [8]. In another study including 304 PMF patients, JAK2V617F mutation was associated with increased risk of death and leukemic transformation [17]. In a series of 199 PMF patients, JAK2V617F mutation had no correlation with survival or leukemic transformation [41]. In another study including 77 PMF patients, the presence of JAK2V617F mutation showed no impact on OS and the risk of leukemic transformation [43]. In our series of 105 PMF patients, JAK2V617F-mutated and -unmutated patients showed no significant difference for the rate of death, leukemic transformation, OS, and LFS. Studies about the impact of JAK2V61F mutation on OS and the risk of leukemic transformation in ET and PMF patients have yielded controversial results. In agreement with previous data, our study demonstrated that DIPSS-plus high risk PMF patients had shorter survival compared to the other risk groups [19]. Moreover, LFS was shorter in DIPSS-plus high risk PMF patients compared to the other risk groups. There is limited data regarding the outcome of Ph-negative MPNs according to the JAK2V617F mutation. In a series of 166 total MPN patients, the rate of death was not different between JAK2V617F-positive and JAK2V617F-negative patients [13]. In our series of 410 Ph-negative MPNs, the rate of death and leukemic transformation, OS, and LFS were similar between JAK2V617F-positive and JAK2V617F-negative subgroups.

In conclusion, our results imply that in a large series of PV patients, JAK2V617F mutation is associated with a higher rate of female patients, lower Hgb level, higher leukocyte and platelet counts, and higher prevalence of thrombosis. In a large series of ET patients, our findings suggest that JAK2V617F mutation is associated with PV-like phenotype with higher Hgb and Hct levels and lower platelet counts. Moreover, our JAK2V617F-negative ET patients displayed a trend towards higher rate of leukemic transformation. In PMF patients, our results point out that JAK2V617F mutation is associated with a more pronounced myeloproliferative phenotype with higher leukocyte count, greater spleen size, a trend towards higher Hgb level. Moreover, the rate of females was lower in JAK2V617F-mutated PMF patients. In a total of very large number of Ph-negative MPN patients, our findings support that JAK2V617F mutation is associated with a more aggressive phenotype witha trend towards older age at diagnosis, higher leukocyte count, higher Hgb and Htc levels and lower platelet count, a trend towards greater spleen size, higher frequency of phlebotomy, a trend towards a higher prevalence of cardiovascular risk factors and thrombosis.

There are limitations that need to be acknowledged and addressed regarding the present study. The first limitation concerns the retrospective nature of the study. Prospective studies are required to confirm the results of the present study. The second limitation concerns the characteristics of the study population of our PV patients. The frequency of JAK2V617F mutation in our PV patients is lower compared to the previous reports (81.5% and 96%, repectively). Since our study population is composed of the patients of a reference center, to which many JAK negative patients are referred and are diagnosed with JAK2V617F-negative PV. Thus, the patient population can be considered to have potential bias and our results cannot be extended to the entire population of PV patients.

The impact of JAK2V617F mutation on clinical phenotype in Ph-negative MPNs is still debated. As a whole, our comprehensive study including large number of Turkish MPN patients may indicate that JAK2V617F mutation is accociated with distinct disease phenotypes of PV, ET, PMF, and Ph-negative MPNs.

## Figures and Tables

**Table 1:** Clinical and laboratory characteristics of patients with Ph-negative myeloproliferative neoplasms according to the JAK2V617F mutation (n = 410).

Ph-negative myeloproliferative neoplasms	JAK2V617F-mutated n (%)	JAK2V617F- unmutated n (%)	p value
Hct at diagnosis (%)	43.46 [9.66]	39.75 [9.42]	0.001
Platelet count at diagnosis (mm^3^)	639.420 [351.700]	799.320 [521.100]	0.003
LDH at diagnosis (U/L)	465.9 [315.3]	438.1 [260.3]	0.876
Spleen size at diagnosis (mm)	148.33 [43.08]	137.74 [31.87]	0.056
Follow-up duration (months)	82.04 [63.07]	74.67 [61.73]	0.224
Number of patients	298	112	-
Risk factors for cardiovascular diseases	223 (74.8%)	73 (65.2%)	0.052
Phlebotomy	105 (35.2%)	23 (20.5%)	0.004
Bleeding	43 (14.4%)	9 (8%)	0.083
Thrombosis	100 (33.6%)	29 (25.9%)	0.059
Leukemic Transformation	9 (3%)	3 (2.7%)	0.855
Death	51 (17.1%)	12 (10.7%)	0.11

**Table 2:** Clinical and laboratory characteristics of patients with essential thrombocythemia patients according to JAK2V617F mutation (n = 170).

ET	JAK2V617F-mutated (mean [SD])	JAK2V617F-unmutated (mean [SD])	p value
Number of patients	108	62	-
Females (%)	70 (64.8%)	32 (51.6%)	0.091
Age at diagnosis	49.74[15.86]	51.15[14.87]	0.506
Age at time of data collection	57.39[16.12]	58.87[14.87]	0.554
Leukocyte at diagnosis (mm^3^)	10.344[3751]	10.119 [3288]	0.819
Hgb at diagnosis (g/dL)	13.86 [1.56]	12.55 [1.96]	0.001
Hct at diagnosis (%)	41.95 [4.90]	37.59 [5.52]	0.001
Platelet count at diagnosis (mm^3^)	860.810 [310.900]	1.057.950 [465.300]	0.001
LDH at diagnosis (U/L)	359.2 [170.4]	400.8 [172.9]	0.078
Spleen size at diagnosis (mm)	132.49 [28.8]	128.51 [20.72]	0.495
Risk factors for cardiovascular diseases	38 (61.3%)	74 (68.5%)	0.339
Bleeding	12 (11.1%)	5 (8.1%)	0.524
Thrombosis	36 (33.3%)	20 (32.3%)	0.886
Leukemic transformation	0	2 (3.2%)	0.061
Death	5 (8.1%)	13 (12%)	0.419

**Table 3:** Clinical and laboratory characteristics of patients with Polycythemia vera patients according to JAK2V617F mutation (n = 135).

PV	JAK2V617F-mutated (mean [SD])	JAK2V617F-unmutated (mean [SD])	p value
Number of patients	110	25	-
Females (%)	44 (40%)	3 (12%)	0.008
Age at diagnosis	56.04[13.90]	50.52[14.15]	0.082
Age at time of data collection	62.25[13.91]	56.68 [14.51]	0.071
Leukocyte at diagnosis (mm^3^)	12.970[5590]	8.538 [2797]	0.001
Hgb at diagnosis (g/dL)	16.59 [2.60]	17.78 [1.94]	0.018
Hct at diagnosis (%)	51.2 [7.67]	52.33 [6.12]	0.622
Platelet count at diagnosis (mm^3^)	557.090 [268.200]	382.330 [355.900]	0.001
LDH at diagnosis (U/L)	375 [220]	294 [110]	0.202
Spleen size at diagnosis (mm)	131 [24]	127 [21]	0.351
Follow-up duration (months)	70.81 [56.76]	68.7 [55.86]	0.883
Risk factors for cardiovascular diseases	92 (83.6%)	22 (88%)	0.764
Phlebotomy	90 (81.8%)	20 (80%)	0.782
Bleeding	12 (10.9%)	2 (8%)	0.499
Thrombosis	47 (42.7%)	5 (20%)	0.035
Leukemic transformation	2 (1.8%)	0	0.499
Death	9 (8.2%)	1 (4%)	0.473

**Table 4:** Clinical and laboratory characteristics of patients with Primary myelofibrosis patients according to JAK2V617F mutation (n = 105).

PMF	JAK2V617F-mutated (mean [SD])	JAK2V617F-unmutated (mean [SD])	p value
Number of patients	80	25	-
Females (%)	35 (43.8%)	21 (84%)	0.001
Age at diagnosis	58.2 [13.80]	52.88 [15.68]	0.107
Age at time of data collection	64.05 [13.17]	60 [14.91]	0.197
Leukocyte at diagnosis (mm^3^)	16.180 [13.865]	10.377 [7771]	0.019
Hgb at diagnosis (g/dL)	11.55 [2.70]	10.54 [2.59]	0.056
Hct at diagnosis (%)	34.85 [8.7]	32.53 [8.01]	0.162
Platelet count at diagnosis (mm^3^)	453.760 [351.700]	574.900 [415.300]	0.224
LDH at diagnosis (U/L)	735 [408]	674 [376]	0.606
Spleen size at diagnosis (mm)	193 [48]	171 [40]	0.042
Follow-up duration (months)	65.74 [58.19]	75.36 [55.32]	0.304
Risk factors for cardiovascular diseases	57 (71.3%)	13 (52%)	0.075
Bleeding	19 (23.8%)	2 (8%)	0.086
Thrombosis	17 (21.3%)	4 (16%)	0.567
Leukemic Transformation	7 (8.7%)	1 (4%)	0.437
Death	29 (36.25%)	6 (24%)	0.259
Risk factors for cardiovascular diseases	57 (71.3%)	13 (52%)	0.075
